# Inhibitors of Human Neuraminidase Enzymes Block Transmigration *in vitro*

**DOI:** 10.3389/fmolb.2022.835757

**Published:** 2022-02-25

**Authors:** Md. Amran Howlader, Tianlin Guo, Christopher W. Cairo

**Affiliations:** Department of Chemistry, University of Alberta, Edmonton, AB, Canada

**Keywords:** neuraminidase, sialidase, sialic acid, cell adhesion, migration, transmigration, chemotaxis

## Abstract

Cell migration to a site of inflammation is an important step of the immune response. This process is coordinated by cytokines, receptors, and the signal processing machinery of the cell. Many cellular receptors are glycosylated, and their activity can be modulated through changes in glycan structure. Furthermore, glycosylation can be critical to the folding and trafficking of receptors. In this work, we investigated the role of native human neuraminidase enzymes (NEU) in transmigration. We used a cultured T cell line (Jurkat) and a transwell assay with fibronectin (FN) coated wells and cytokines (IL-4 and TNF-α) as chemoattractants in the bottom chamber. We observed that NEU1, NEU3, and NEU4 were positive regulators of transmigration using an siRNA knockdown. Furthermore, we found that pharmacological inhibition of these enzymes inhibited transmigration. We conclude that human NEU isoenzymes NEU1, NEU3, and NEU4 can act as positive regulators of transmigration and should be investigated as targets for anti-inflammatory strategies.

## Introduction

The migration of leukocytes to a site of inflammation is an important step in the inflammatory cascade (Ley et al., 2007). Leukocytes can become activated upon stimulation by cytokines and engage a series of receptors in peripheral tissue that allow them to home to the site of inflammation (Springer, 1994; Johnston and Butcher, 2002). Importantly, most of these receptors are glycosylated, and changes to their glycan structures may regulate immune function (Rudd et al., 1999; Marth and Grewal, 2008). Many glycan structures are terminated by *N*-acetyl-neuraminic acid (sialic acid), and the presence of this residue can be critical for protein-glycan recognition. A primary example of this phenomenon is the sialyl-Lewis x antigen (CD15s). The CD15s antigen acts as a selectin ligand, but loss of the sialic acid from the antigen (forming CD15) prevents selectin binding (Tiemeyer et al., 1991). Thus, regulation of sialyation is critical to such interactions (Gadhoum and Sackstein, 2008). Sialic acid residues are installed by sialyltransferase and removed by neuraminidase (NEU; or sialidase) enzymes (Varki et al., 2015).

There are four isoenzymes of human NEU, NEU1-4, which have distinct subcellular and tissue expression and substrate preferences (Miyagi and Yamaguchi, 2012). The NEU1 enzyme hydrolyzes *N*-acetyl-neuraminic acids (sialic acids) from proteins, while NEU3 acts primarily on glycolipids. NEU4 can modify both glycoproteins and glycolipids (Wang et al., 2001; Seyrantepe et al., 2004; Sandbhor et al., 2011; Smutova et al., 2014). The human NEU enzymes have a growing list of potential functions in the immune system; some examples include: removal of leukocyte selectin ligands (Gadhoum and Sackstein, 2008), regulation of toll-like receptors (Amith et al., 2010), regulation of cytokine production (Stamatos et al., 2010), modulation of integrin adhesion (Howlader et al., 2019), and regulation of LDL uptake by macrophages in atherosclerosis (Demina et al., 2021).

Our understanding of the role of human NEU in the regulation of cell adhesion and migration is only beginning to emerge. In previous studies, we have investigated the influence of human NEU on the migration of adherent cells on a FN substrate through the β1-integrin (Howlader et al., 2020). In those studies, we observed distinct roles for isoenzymes: NEU1 was a negative regulator of migration, while NEU3 and NEU4 were positive regulators. We reasoned that a similar difference in NEU function in lymphocytes could have important implications for these enzymes in anti-inflammatory or wound-healing applications. Here, we investigated the influence of human NEU isoenzymes NEU1, NEU3, and NEU4 on β1-integrin dependent transmigration in a lymphocyte model (Jurkat T cells). We used siRNA to test the role of native expression of the enzymes, and small molecule inhibitors to test their role pharmacologically. We find that NEU1, NEU3, and NEU4 act as positive regulators of transmigration, suggesting these enzymes could be novel anti-inflammatory targets.

## Materials and Methods

### Materials

NEU1 siRNA, NEU3 siRNA, and scRNA was procured from Dharmacon, United States (see Supplementary Table S1 for sequences). Lipofectamine 2000 was procured from Thremofisher, United States. Rabbit anti-human β-actin antibody (ab8227) was procured from Abcam, United States. HRP-conjugated Goat anti-rabbit and goat anti-mouse antibodies were procured from Biorad, United States. Tumor Necrosis Factor-α (TNF-α), Interleukin-4 (IL-4), Streptavidin-HRP, and mouse anti-human NEU1 antibody was procured from R&D systems, United States. Mouse anti-human NEU3 antibody (Clone 11B) was procured from MBL international (Woburn, MA, United States). Fibronectin (FN) was procured from Calbiochem, United States. *Maackia amurensis* (MAL) and Sambucus Nigra (SNA) lectins were procured from bio-World, United States. Jurkat T cells (Clone E6.1) were procured from ATCC (Manassas, VA, United States). Inhibitors CG14600 **1**, C9-BA-DANA **2**, CG22600 **3**, CZ53100 **4**, CY16600 **5**, and DANA **6** were prepared and characterized as previously reported (Zou et al., 2010; Zhang et al., 2013; Guo et al., 2018a; Guo et al., 2018b).

### Transfection of Jurkat T cells With siRNA

Briefly, siRNA (20 µL) and lipofectamine 2000 (10 µL) was mixed in serum-free RPMI medium. Then Jurkat T cells (5 × 10^6^) were treated with NEU1 siRNA, NEU3 siRNA, or a scrambled control (scRNA) mixture for 72 h following the manufacturer’s protocol (Thermofisher, United States) without antibiotics. After incubation, cells were collected and lysed using RIPA buffer. Protein concentrations were measured using a BCA assay and equal amounts of protein were loaded in each lane of a gel used for Western blots. Western blots for NEU1 used a mouse anti-human NEU1 antibody (1:5000 dilution); and for NEU3 used mouse anti-human NEU3 antibody (1:2000 dilution). HRP-conjugated goat anti-mouse antibody was used as a secondary antibody. The blots were developed using enhanced chemiluminescence reagent (ECL, Biorad, United States) (Supplementary Figures S2–S4). As a secondary check for protein loading, we performed a separate blot for β-actin. The primary β-actin antibody was used at 1:5000 dilution, and secondary antibody was used at 1:3000 dilution.

### Transwell Migration Assays

Transwell migration assays were performed following protocols from Justus et al. (2014), with slight modifications for Jurkat T cells. Jurkat T cells (250 μL, 1 × 10^4^ cells well^−1^) were carefully spread on top of a FN-treated 3 µm pore upper chamber of a Costar transwell plate (Corning, United States). The upper chamber was maintained with serum-reduced RPMI medium. Inhibitors were applied on both the upper and the lower chambers at the indicated concentration. The bottom chamber was loaded with media containing cytokines (IL-4, 20 ng/ml^−1^; TNF-α, 10 ng ml^−1^) and 10% FBS in RPMI media as a chemoattractant (Leung et al., 1994; Xia et al., 1996). Serum-free RPMI was used in the bottom chamber as a negative control. Cells were allowed to transmigrate for 21 h at 37°C in a humidified incubator with 5% CO_2_. Cells were then collected from the bottom chamber and counted using a flow cytometer (BD Accuri C6, BD Biosciences). Normalized transmigration was calculated using the formula: Normalized transmigration, $Tr=\left(\frac{\text{N}21}{N_{B}}\right)\times 100$ . Where N_21_ was the number of live cells per 50 µL of medium in the bottom chamber after 21 h, and N_B_ was the number of live T cells per 50 µL of medium in the bottom chamber of the control well.

### Cell Viability Assays

Viability of cells after treatment was assayed under similar conditions that were used in the transmigration studies. Briefly, wells of a 96 well plate were charged with 100 µL of 50 × 10^4^ cells mL^−1^ and incubated for 18 h at 37°C in a humidified incubator with 5% CO_2_. Cells were then treated with the indicated conditions for 21 h. The final concentration of the compounds was the same as that used for transmigration studies. After incubation, 20 µL of MTS solution (Promega, United States) was added to each well and incubated for 2 h. The plate was spun at 200 x g for 5 min to settle the cells on the bottom of the plate. The absorbance of soluble formazan produced by viable cells from metabolism of MTS was measured at 490 nm using SpectraMax M2 (Molecular Devices, United States) plate reader. For each condition, the experiment was conducted with replicates on different days to consider the intra- and inter-day variabilities. Absorbance for each replicate was normalized to that of an intra-day buffer control.

## Results

### NEU1 and NEU3 Expression Levels Were Reduced in Jurkat T cells Using siRNA

**FIGURE 1:**
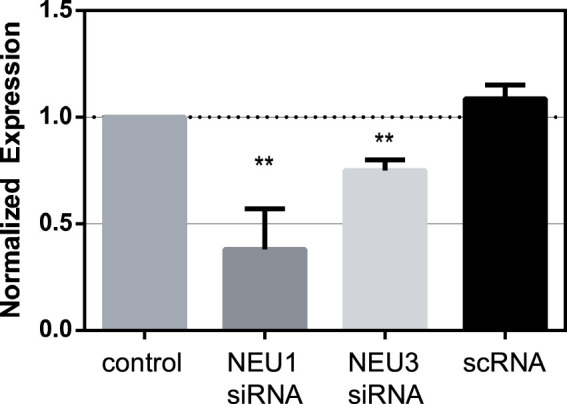
Knockdown of NEU1 and NEU3 expression using siRNA. Jurkat cells were transfected with siRNA for NEU1, NEU3, or with a scrambled control (scRNA). Western blots were used to determine changes in expression of NEU1 and NEU3 (see Supporting Information). Blots were analyzed by densitometry, and the average changes in expression are shown relative to controls. Values were compared to control using a Student’s t-test (**p < 0.01).

Before investigating the activity of NEU enzyme inhibitors, we sought to determine the role of native NEU expression in transmigration. We selected Jurkat T cells as a reproducible and stable T cell line for our experiments. The expression of NEU1, NEU3, and NEU4 has been altered through transfection of cells with siRNA (Howlader et al., 2020). Using siRNA, we were able to significantly reduce the expression levels of NEU1 by ∼60% and that of NEU3 by ∼20% (Figure 1; Supplementary Figures S2–S4). Unfortunately, we were unable to measure changes in NEU4 expression levels as we were unable to find a suitable antibody for Western blots (Howlader et al., 2020). We used an identical protocol for siRNA knockdown of NEU1, NEU3, and NEU4 to investigate the influence of these enzymes on T cell transmigration.

### Interference with NEU1, NEU3, and NEU4 Expression Reduced Transmigration

**FIGURE 2:**
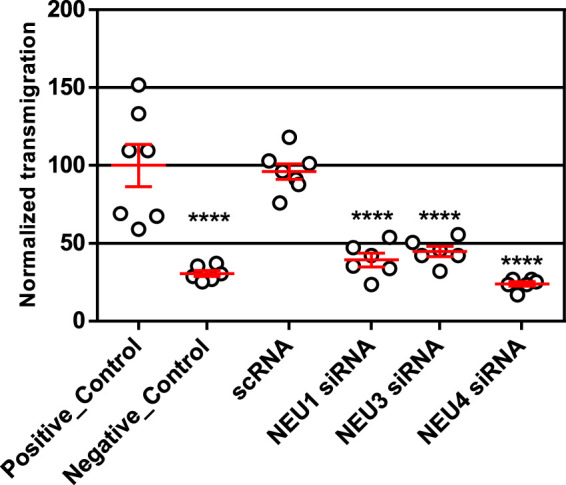
Transmigration of cells after NEU1, NEU3, and NEU4 knockdown. Transmigration experiments were carried out with a FN-coated transwell plate 21 h after knockdown treatment. The bottom chamber was loaded with serum and cytokine-free media as an untreated control (positive control); CytoD was used as a negative control for transmigration. All other experiments used a lower chamber containing cytokine and serum as attractant. Cells were allowed to transmigrate for 21 h and were counted by flow cytometry. Data shown are for six (Tiemeyer et al., 1991) replicates (3 replicates over two experiments); represented as mean ± SEM and compared to scrambled control using a One-way ANOVA followed by Dunnett’s post-hoc analysis (****p ≤ 0.001).

We implemented a transwell assay to investigate the effects of NEU isoenzyme expression on Jurkat T cell transmigration (Justus et al., 2014). Cells were seeded into the top chamber of a transwell plate, where the filter was pre-treated with fibronectin (FN). The bottom chamber contained media with serum and inflammatory cytokines as chemoattractants (TNF-α and IL-4) (Leung et al., 1994; Xia et al., 1996). The bottom chamber was loaded with serum- and cytokine-free media for negative controls. Cells were pre-treated with scRNA or siRNA for NEU knockdown of the relevant enzyme before being seeded into the transwell chamber. Once loaded into the chamber, cells were allowed to migrate for 21 h, collected from the bottom chamber and then counted by flow cytometry. We observed that cytokines were required to obtain significant chemotaxis of the cells. Compared to scRNA controls, we observed that siRNA for NEU1, NEU3, and NEU4 significantly inhibited transmigration (Figure 2; Supplementary Table S2). We concluded that NEU1, NEU3, and NEU4 act as positive regulators of transmigration. As noted above, we could not confirm a change in expression level for NEU4; however, these results are consistent with pharmacological inhibition (*vide infra*) (Howlader et al., 2020).

### Inhibitors of NEU1, NEU3, and NEU4 Block Transmigration

**FIGURE 3:**
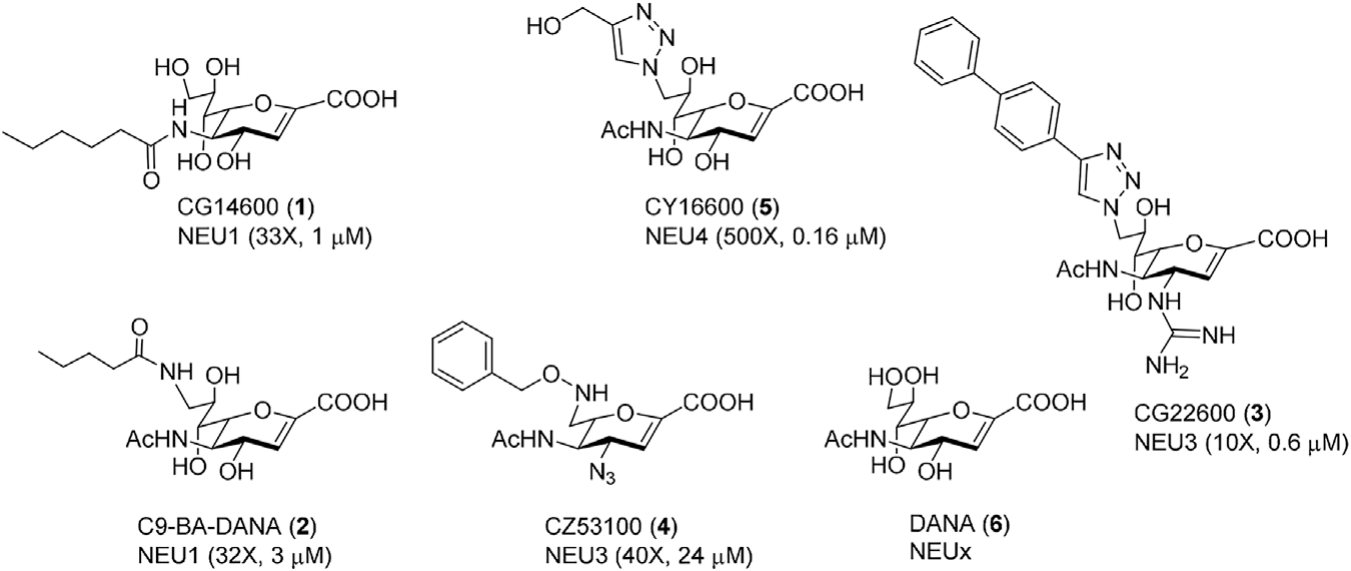
Selective human NEU inhibitors used in this study. Inhibitors were selected from previous reports for selectivity against different human NEU isoenzymes. Each compound is shown with a unique identifier and a compound number used in this work. The isoenzyme which the inhibitor targets is given, along with the fold-selectivity (as compared to its next-best NEU target), along with its reported IC_50_ value. Compound **1** (Guo et al., 2018a) and **2** (Magesh et al., 2008) are selective NEU1 inhibitors. Compound **3** (Guo et al., 2018b) and **4** (Zhang et al., 2013) are selective NEU3 inhibitors. Compound **5** (Albohy et al., 2013) is a selective NEU4 inhibitor. Compound **6** is a general inhibitor with activity for all human NEU isoenzymes (Richards et al., 2018).

**FIGURE 4:**
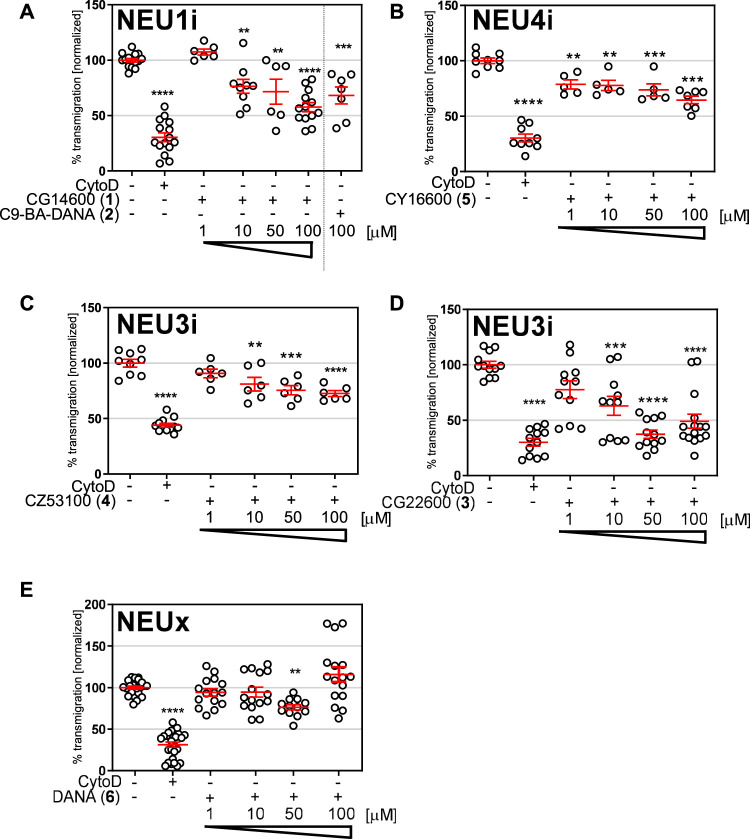
Transmigration after treatment with NEU inhibitors. Transmigration experiments were carried out with a FN-coated transwell plate. The bottom chamber was loaded with serum and cytokine-free media as an untreated control; CytoD was used as a negative control for transmigration. All other experiments used a lower chamber containing cytokine and serum as attractant. Cells were loaded in the top well with the indicated concentration of inhibitors for **(A)** NEU1, **(B)** NEU4, **(C,D)** NEU3, or **(E)** non-specific inhibitor DANA **6**. Cells were allowed to transmigrate for 21 h and were counted by flow cytometry. Data shown for inhibitors include six technical replicates and two biological replicates; and are represented as mean ± SEM and compared to control using one-way ANOVA followed by Dunnett’s post-hoc analysis (****p ≤ 0.001; ***p < 0.005; **p < 0.01).

Our group (Albohy et al., 2013; Cairo, 2014; Guo et al., 2018a; Guo et al., 2018b), and others (Magesh et al., 2008), have reported the development of selective inhibitors of the human NEU enzymes. Interestingly, widely known inhibitors of viral NEU enzymes are not very active or selective against the human enzymes (Hata et al., 2008; Richards et al., 2018). For this study, we identified a set of inhibitors with variable selectivity for NEU1, NEU3, and NEU4 to investigate for effects on transmigration (Figure 3). Compounds were tested over a concentration range of 1–100 μM in transmigration assays using wild-type Jurkat T cells. For these experiments, cytochalasin D (cytoD) was used as a negative control for cell transmigration. CytoD acts by disruption of the actin cytoskeleton by stopping the polymerization of actin monomers (Wakatsuki et al., 2001). Since the structure of the actin cytoskeleton is essential for cell movement, CytoD is a potent inhibitor of cell migration. The transwell assay again used serum and cytokines as chemoattractants in the bottom chamber, and cells were seeded into the top chamber with the indicated concentration of inhibitor (Figure 4).

We tested two inhibitors of NEU1, CG14600 **1** and C9-BA-DANA **2**. Both compounds are reported to have ∼30-fold selectivity for NEU1 (Magesh et al., 2008; Guo et al., 2018a. We observed that both compounds were active as inhibitors of transmigration at the highest concentration tested (100 µM). Between these, CG14600 **1** treatment showed a more potent response with significant inhibition at concentrations as low as 10 µM. Compound CY16600 **5** has 500-fold selectivity for NEU4 and is the most potent human NEU inhibitor reported to date (Albohy et al., 2013). When tested in the transwell assay, we observed a dose-dependent inhibition of migration with significant effects at 1–100 µM. We tested two inhibitors of NEU3, CZ53100 **4** and CG22600 **3**, which differ in both their potency and selectivity (Figure 3; Supplementary Table S3) (Zhang et al., 2013; Guo et al., 2018b). Both the compound CZ53100 **4** and CG22600 **3** showed significant inhibition of migration at the 10–100 µM concentration. Although CG22600 **3** showed inhibition at concentrations as low as 10 μM, the highest concentration showed more than 50% inhibition of migration. Finally, to provide a point of comparison, we tested a non-specific inhibitor of NEU enzymes, DANA **6**. As an inhibitor, DANA has activity against all four human isoenzymes with some preference for NEU3 and NEU4 (Richards et al., 2018). We observed moderate inhibition of migration for DANA at 50 μM; however, higher concentrations did not show significant activity. We speculate that the mixed activity of DANA is a result of its non-specific effects on different NEU isoenzymes (Howlader et al., 2020).

### Inhibitors of Human NEU Enzymes did Not Reduce the Viability of cells

**FIGURE 5:**
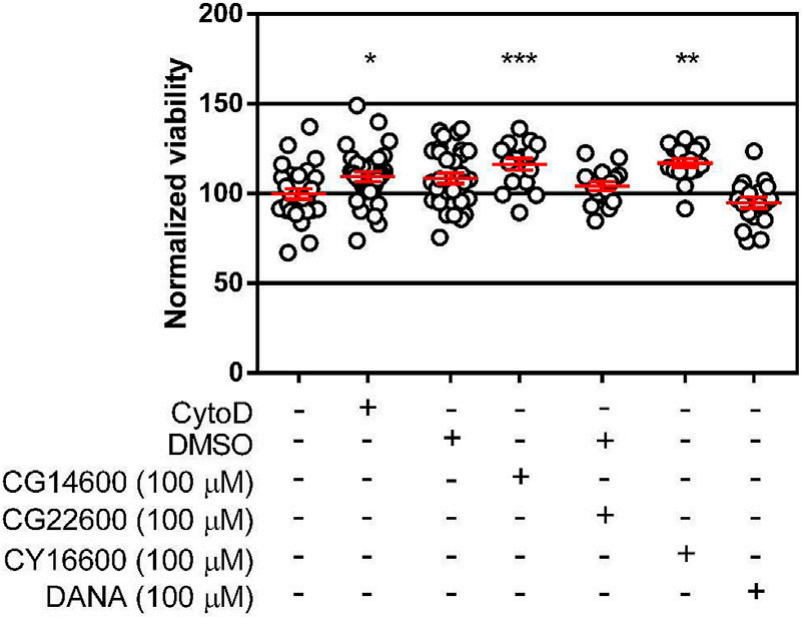
Viability of cells after treatment with inhibitors. Viability of cells was tested after treatment with the highest concentrations of inhibitors used for transmigration studies. Jurkat T cells (5 × 10^4^ cells) were incubated in each well of a clear 96-well plate with the indicated inhibitors for 21 h. After incubation, MTS was used to determine cell viability after 1 h incubation. Data are plotted as the mean ± SEM and compared to control using a Dunnett’s *t*-test (*p ≤ 0.05; **p ≤ 0.01; ***p ≤ 0.005).

A possible explanation for reduced transmigration of cells in the presence of inhibitors is toxicity of the compounds. To test this hypothesis, we evaluated the cytotoxicity of compounds CG14600 **1,** CG22600 **3,** CY16600 **5,** and DANA **6** in Jurkat T cells (Figure 5; Supplementary Table S4). Compounds were tested under identical conditions used in cell migration experiments, with the highest concentration used for each compound. We did not observe any significant cytotoxicity for the inhibitors tested. Compounds CG14600 **1** and CY16600 **5** showed slightly increased viability. Treatment of cells with the cytokines used as chemoattractants (IL-4 and TNF-α) had no effect on T cell viability (Supplementary Figure S1; Supplementary Table S5). From these experiments, we concluded that the cytotoxicity of the inhibitors was not responsible for the observed effects on transmigration.

## Discussion

In this study, we examined the role of native human NEU isoenzymes in transmigration. We show that reduced expression of NEU1, NEU3, and NEU4 attenuated transmigration, suggesting these enzymes could be considered positive regulators of transmigration. We then tested the effects of specific inhibitors of human NEU isoenzymes on transmigration on FN-treated transwell inserts using TNF-α and IL-4 as chemoattractant. In agreement with our siRNA results, we observed that selective inhibitors of human NEU1, NEU3, and NEU4 could inhibit transmigration. Among these, CG22600 **3** showed the greatest potency. We also tested the pan-selective NEU inhibitor, DANA **6**, which was found to be a weak inhibitor of transmigration. Finally, we confirmed that the activity of our compounds was not due to cytotoxicity. Together, our results establish a role for human neuraminidase enzymes in the regulation of cell migration and may suggest these enzymes as targets for the development of anti-inflammatory therapeutics.

The glycosylation of extracellular receptors plays a critical role in cell migration and adhesion. Here, we examined the influence of human NEU enzyme activity on a lymphocyte migration model dependent on β1-integrin-FN interactions. Glycosylation of integrin receptors is known to affect adhesion (Bellis, 2004; Schultz et al., 2012), and we sought to investigate if NEU enzymes could regulate transmigration in lymphocytes. We have previously found that NEU3 and NEU4 can modify β1-integrin *in vitro*, and that NEU activity may affect integrin endocytosis (Howlader et al., 2020). Our siRNA experiments (Figure 2) establish that NEU1 and NEU3 act as positive regulators of transmigration in our model. The data are also consistent with NEU4 acting as a positive regulator, but we could not confirm changes in NEU4 expression. We also caution that our results used only one cell type (Jurkat T cells) and chemoattractant with an *in vitro* model. Future work is planned to investigate if these effects are consistent in primary leukocytes.

Although inhibitors of NEU enzymes are well known in antiviral therapy, clinically approved viral NEU inhibitors have limited activity and specificity against human NEU (Hata et al., 2008; Richards et al., 2018). The presence of four isoenzymes of NEU in humans presents a possibility for off-target effects with non-specific NEU inhibitors. These factors can complicate the interpretation of results for human NEU when using DANA **6**, oseltamivir, zanamivir, or other antivirals. In recent years, inhibitors selective for individual human NEU isoenzymes have been developed in recognition of this limitation (Magesh et al., 2008; Zhang et al., 2013; Albohy et al., 2013; Cairo, 2014; Guo et al., 2018a; Guo et al., 2018b). These compounds allowed us to interrogate NEU1, NEU3, and NEU4 pharmacologically in transmigration (Figure 4). We found that inhibitors of NEU1, NEU3, and NEU4 all acted as inhibitors of transmigration. The most potent of these were compounds specific for NEU3. In previous studies, we have observed that inhibitors that target different isoenzymes can compete with each other, resulting in masking the role of individual NEU isoenzymes (Howlader et al., 2020). This effect may explain why DANA **6** showed only marginal activity as an inhibitor in our transmigration assays. We also cannot rule out whether differences in the physical properties of these compounds or their recognition by transporters could play a role in these observed differences.

Sialoglycoproteins and glycolipids play a variety of roles in cell biology, and the regulation of sialylation may influence recognition and signaling processes. Sialosides participate in regulating T cell activation, and targeted degradation of sialosides is emerging as a strategy for immunotherapy (Gray et al., 2020; Edgar et al., 2021). Sialic acids have been found to mediate a large number of glycoprotein interactions, including adhesion receptors such as the β1-integrin, a receptor for FN (Johansson et al., 1997; Schultz et al., 2012; Xie et al., 2021). In cancers that overexpress NEU1 the enzyme has been found to act as a negative regulator of migration through disruption of β1-and β4-integrin interactions (Uemura et al., 2009; Zhou et al., 2020). In other cancer models, NEU1 has been found to be a positive regulator of cell migration (Hou et al., 2016; Ren et al., 2016). NEU1 has also been found to be a positive regulator of lymphocyte infiltration in pulmonary fibrosis models (Luzina et al., 2016; Luzina et al., 2020). Similarly, NEU3 has been found to act as a positive regulator of β1-integrin-mediated cell migration in renal cancer cells (Tringali et al., 2012). Our transmigration model found that all NEU isoenzymes tested acted as positive regulators (NEU1, NEU3, and NEU4). It is likely that extracellular matrix glycoproteins and specific sialoglycoconjugates are critical to the function of NEU enzymes during cell migration *in vivo*. We previously observed that NanI and NEU3 can alter sialylation of LFA-1 (a β2-integrin) *in vitro* (Howlader et al., 2019), and that NEU enzymes and inhibitors can alter the endocytosis of integrins (Howlader et al., 2019; Howlader et al., 2020). In experiments examining cell migration on a FN-coated surface, we found that pre-treatment of FN with NEU did not affect rates of migration (Jia et al., 2016). Thus, we speculate that NEU enzyme activity regulates cell migration through changes to sialoglycoconjugates on the cell surface, such as integrins or glycolipids.

Indeed, NEU isoenzymes have context-dependent effects on cell adhesion, varying by cell type and receptor system. Using an *in vitro* model of adherent cell migration with a FN substrate, we previously reported that NEU3 and NEU4 act as positive regulators of migration, while NEU1 acted as a negative regulator (in PC-3 and MBA-MD-231 cells) (Howlader et al., 2020). In the case of NEU3 and NEU4, these enzymes have activity for glycolipid substrates which may provide a mechanism for their effects (Jia et al., 2016). Furthermore, we have observed that NEU3 acts as a negative regulator of β2-integrin-mediated static adhesion while acting as a positive regulator of β1-integrin-mediated homotypic aggregation in lymphocytes (Howlader et al., 2019). We propose that using isoenzyme-selective inhibitors of NEU provides new opportunities for understanding the complex role of sialoglycoconjugates and the enzymes that modify them in inflammation and cell migration.

Using an *in vitro* model of T cell transmigration, we observed that native activity of NEU1 and NEU3 act as positive regulators. Our data support that NEU4 also acts as a positive regulator in this model, but we could not confirm expression levels for this isoenzyme. Treatment of cells with selective inhibitors of human NEU isoenzymes allowed us to confirm these effects in the same assay. Small molecule inhibitors selective for NEU1, NEU3, and NEU4 all acted as inhibitors of transmigration. Compounds with mixed selectivity, such as DANA **6**, showed less consistent inhibition with reduced potency as compared to selective inhibitors. The activity of selective human NEU inhibitors should be investigated in whole animal models and with different cell types to establish the role of NEU enzymes in inflammation.

## Data Availability

The original contributions presented in the study are included in the article/Supplementary Material, further inquiries can be directed to the corresponding author.
