# Supplementary material for: Inhibitors of Human Neuraminidase Enzymes Block Transmigration in vitro
Source: Front Mol Biosci. 2022 Feb 25;9:835757. doi: 10.3389/fmolb.2022.835757 (PMC8913934; doi:10.3389/fmolb.2022.835757)
Supplement: Supplementary file 1 [file DataSheet1.PDF]

Supporting information for:

Inhibitors of human neuraminidase enzymes block transmigration in vitro

Md. Amran Howlader, Tianlin Guo, and Christopher W. Cairo*

Department of Chemistry, University of Alberta, Edmonton Alberta, T6G 2G2, Canada

*To whom correspondence should be addressed. Tel.: 780 492 0377; fax: 780 492 8231; e-mail: ccairo@ualberta.ca

Table of contents:

Table S1: Sequences of scrambeled, NEU3, and NEU1 siRNA 2
Table S2: Normalized transmigration of NEU1 and NEU3 knockdown cells 3
Table S3: Normalized transmigration of inhibitor-treated cells 4
Table S4: Viability of cells treated with inhibitors... 5
Table S5: Viability of cells treated with IL-4 and TNF-α 5
Figure S1: Viability of cells after treatment with cytokines. 6
Figure S2: Expression of NEU1 after siRNA knockdown. 7
Figure S3: Expression of NEU3 after siRNA knockdown. 8
Figure S4: Summary of siRNA knockdown experiments for NEU1 and NEU3................ 9

Table S1: Sequences of scrambeled, NEU3, and NEU1 siRNA

ON-TARGETplus Non-targeting siRNA #1 sequence (catalog # D-001810-01-05)

Target sequence:	UGGUUUACAUGUCGACUAA

NEU3 sequences (catalog # L-010641-02-0005)

Target sequence:	ACUGGAUAAUAGUGCGUAU
Antisense:	AUACGCACUAUUAUCCAGU
Target sequence:	CUCAUUAGGCCCAUGGUUA
Antisense:	UAACCAUGGGCCUAAUGAG
Target sequence:	GGGCCAUGUCACAGAGCGU
Antisense:	ACGCUCUGUGACAUGGCCC
Target sequence:	CUGUUUACACACCGGGAGA
Antisense:	UCUCCCGGUGUGUAAACAG

NEU4 sequences (catalog # L-013263-01-0005)

Target sequence:	GUGCAGAUCGCCACGGGAA
Antisense:	UUCCCGUGGCGAUCUGCAC
Target sequence:	GUGAGGUCCUGGAGAACGU
Antisense:	ACGUUCUCCAGGACCUCAC
Target sequence:	AAAUAAAGGAAUCGUGCUU
Antisense:	AAGCACGAUUCCUUUAUUU
Target sequence:	AGGGCAGCAUCGUGGGCUU
Antisense:	AAGCCCACGAUGCUGCCCU

NEU1 sequences (catalog # L-011092-00-0005)

Target sequence:	GGCAGCACAUGGUCUCCUA
Antisense:	UAGGAGACCAUGUGCUGCC
Target sequence:	AGUGAGCGAUGUUGAGACA
Antisense:	UGUCUCAACAUCGCUCACU
Target sequence:	GAACGACUUCGGUCUGGUG
Antisense:	CACCAGACCGAAGUCGUUC
Target sequence:	GAGCAAGGAUGAUGGUGUU
Antisense:	AACACCAUCAUCCUUGCUC

Table S2: Normalized transmigration of NEU1 and NEU3 knockdown cells

Condition	N	Transmigration		
		Mean ± SEM		
**Positive Control**	7	100	±	14
**Negative Control CytoD (197 nM)**	6	32	±	2
**NEU1 siRNA**	6	39	±	4
**NEU3 siRNA**	6	45	±	3
**NEU4 siRNA**	6	24	±	2
**scRNA**	6	96	±	5

Table S3: Normalized transmigration of inhibitor-treated cells

Condition	N	Transmigration Mean ± SEM
**Control**	26	100 ± 2
**Cyto D (197 nM)**	30	32 ± 3
**Control (DMSO)**	3	100 ± 2
**1 (100 μM)**	13	57 ± 4
**1 (50 μM)**	6	71 ± 10
**1 (10 μM)**	9	76 ± 6
**1 (1 μM)**	6	107 ± 3
**2 (100 μM)**	7	68 ± 8
**3 (100 μM)**	9	40 ± 3
**3 (50 μM)**	9	40 ± 5
**3 (10 μM)**	8	75 ± 9
**3 (1 μM)**	8	91 ± 6
**3 (100 nM)**	8	101 ± 9
**4 (100 μM)**	6	73 ± 3
**4 (50 μM)**	6	76 ± 4
**4 (10 μM)**	6	81 ± 6
**4 (1 μM)**	6	91 ± 4
**5 (100 μM)**	7	65 ± 3
**5 (50 μM)**	5	74 ± 5
**5 (10 μM)**	5	78 ± 5
**5 (1 μM)**	5	79 ± 4
**6 (100 μM)**	16	116 ± 9
**6 (50 μM)**	12	76 ± 3
**6 (10 μM)**	15	95 ± 6
**6 (1 μM)**	15	94 ± 4

Table S4: Viability of cells treated with inhibitors

Condition	N	Viability		
		Mean±SEM		
**Control**	28	100	±	3
**CytoD (197 nM)**	32	109	±	3
**Control (DMSO)**	32	108	±	3
**CG14600 (100 μM)**	16	116	±	3
**CG22600 (100 μM)**	16	104	±	3
**CY16600 (100 μM)**	16	117	±	2
**DANA (100 μM)**	18	95	±	3

Table S5: Viability of cells treated with IL-4 and TNF-α

Condition	N	Viability		
		Mean±SEM		
**Control**	8	100	±	6
**IL-4 (20 ng/mL)**	8	97	±	7
**TNF-α (10 ng/mL)**	8	103	±	7
**IL-4 + TNF-α**	8	110	±	6

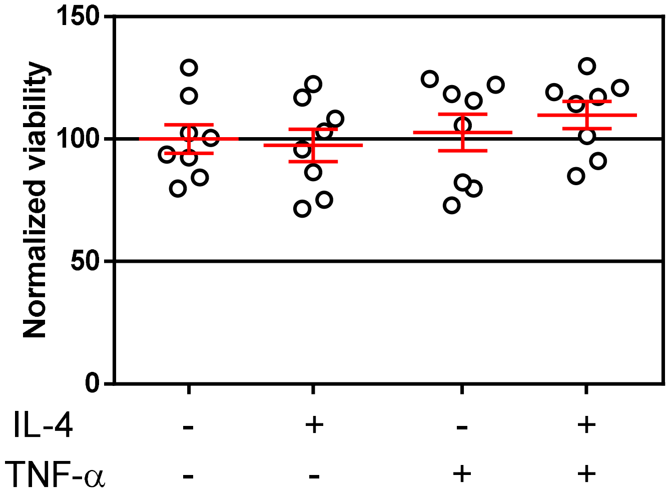

Figure S1: Viability of cells after treatment with cytokines. Viability of Jurkat T cells was carried out after treatment with the IL-4 (20 ng/mL) and TNF-α (10 ng/mL) used for transmigration studies. For each cell line, 5 x 10^4 cells were incubated in each well of a clear 96 plate in the presence of inhibitors for 21 h. After incubation, 20 µl of MTS solution was added to each well and incubated for 1 hour. The absorbance of soluble formazan product was measured at 490 nm using a plate reader (Molecular devices) and viability of the cells were calculated. Data are plotted as the mean ± SEM and compared to control using a Dunnett's t-test (*, $p \leq 0.05$).

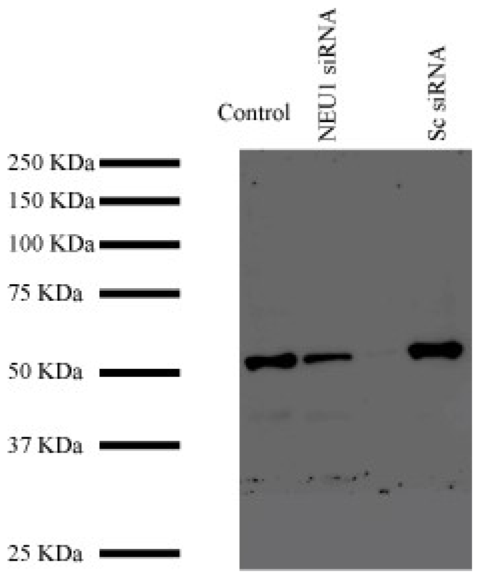

Figure S2: Expression of NEU1 after siRNA knockdown. Representative western blot of NEU1 knockdown of Jurkat T cells. The cells were treated with siRNA or scRNA for 72 h in serum reduced conditions in RPMI. Lysates were collected and equal amount of protein was loaded on SDS-PAGE gel. The blots were developed using mouse anti-human NEU1 antibody as primary and HRP conjugated goat anti-mouse antibody as secondary.

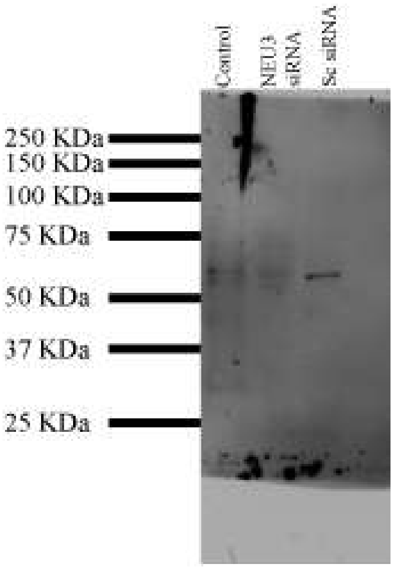

Figure S3: Expression of NEU3 after siRNA knockdown. Representative western blot of NEU1 knockdown of Jurkat T cells. The cells were treated with siRNA or scRNA for 72 h in serum reduced conditions in RPMI. Lysates were collected and equal amount of protein was loaded on SDS-PAGE gel. The blots were developed using mouse anti-human NEU3 antibody as primary and HRP conjugated goat anti-mouse antibody as secondary.

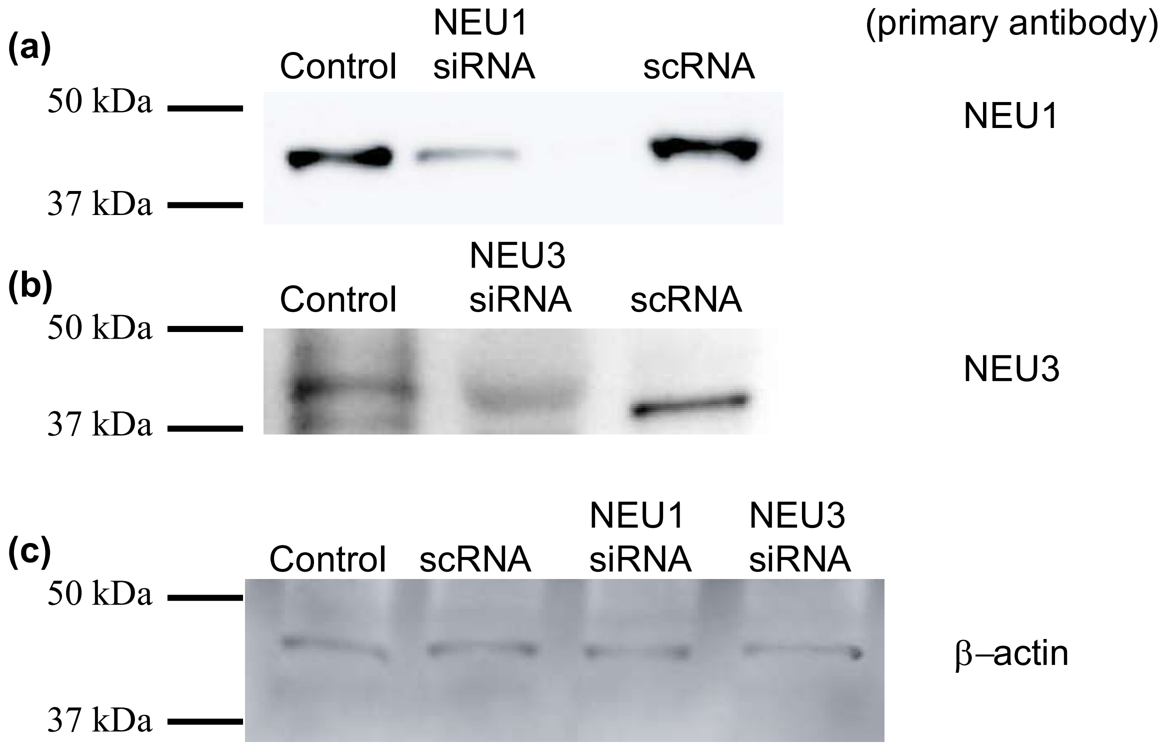

Figure S4: Summary of siRNA knockdown experiments for NEU1 and NEU3. Representative sections of western blots from Jurkat T cell lysate are shown after NEU1, NEU3, or scRNA treatment for 72 h following the manufacturer's protocol (Thermofisher, USA) without antibiotics. After incubation, cells were collected and lysed using RIPA buffer. The blots were developed using mouse anti-human NEU1, anti-human NEU3, or anti-human β-actin antibody as primary and HRP conjugated goat anti-mouse antibody as secondary. Full images for panels A and B are provided in Figures S2 & S3.
